# Soil Bacterial Community Was Changed after Brassicaceous Seed Meal Application for Suppression of *Fusarium* Wilt on Pepper

**DOI:** 10.3389/fmicb.2018.00185

**Published:** 2018-02-13

**Authors:** Gaidi Ren, Yan Ma, Dejie Guo, Terry J. Gentry, Ping Hu, Elizabeth A. Pierson, Mengmeng Gu

**Affiliations:** ^1^Institute of Agricultural Resources and Environment, Jiangsu Academy of Agricultural Sciences, Nanjing, China; ^2^Key Laboratory of Agro-Environment in Downstream of Yangtze Plain, Ministry of Agriculture, Nanjing, China; ^3^Key Lab of Food Quality and Safety of Jiangsu Province–State Key Laboratory Breeding Base, Nanjing, China; ^4^Department of Soil and Crop Sciences, Texas A&M University, College Station, TX, United States; ^5^Department of Horticultural Sciences, Texas A&M University, College Station, TX, United States

**Keywords:** sustainable agricultural systems, Brassicaceous seed meal, *Fusarium* wilt, bacterial community, 454 pyrosequencing

## Abstract

Application of Brassicaceous seed meal (BSM) is a promising biologically based disease-control practice but BSM could directly and indirectly also affect the non-target bacterial communities, including the beneficial populations. Understanding the bacterial response to BSM at the community level is of great significance for directing plant disease management through the manipulation of resident bacterial communities. *Fusarium* wilt is a devastating disease on pepper. However, little is known about the response of bacterial communities, especially the rhizosphere bacterial community, to BSM application to soil heavily infested with *Fusarium* wilt pathogen and cropped with peppers. In this study, a 25-day microcosm incubation of a natural *Fusarium* wilt pathogen-infested soil supplemented with three BSMs, i.e., *Camelina sativa* ‘Crantz’ (CAME), *Brassica juncea* ‘Pacific Gold’ (PG), and a mixture of PG and *Sinapis alba* cv. ‘IdaGold’ (IG) (PG+IG, 1:1 ratio), was performed. Then, a further 35-day pot experiment was established with pepper plants growing in the BSM treated soils. The changes in the bacterial community in the soil after 25 days of incubation and changes in the rhizosphere after an additional 35 days of pepper growth were investigated by 454 pyrosequencing technique. The results show that the application of PG and PG+IG reduced the disease index by 100% and 72.8%, respectively, after 35 days of pepper growth, while the application of CAME did not have an evident suppressive effect. All BSM treatments altered the bacterial community structure and decreased the bacterial richness and diversity after 25 days of incubation, although this effect was weakened after an additional 35 days of pepper growth. At the phylum/class and the genus levels, the changes in specific bacterial populations resulting from the PG and PG+IG treatments, especially the significant increase in Actinobacteria-affiliated *Streptomyces* and an unclassified genus and the significant decrease in Chloroflexi, were suspected to be one of the microbial mechanisms involved in PG-containing BSM-induced disease suppression. This study is helpful for our understanding of the mechanisms that lead to contrasting plant disease severity after the addition of different BSMs.

## Introduction

Growth in sustainable agricultural production systems has generated unprecedented demand for environmentally friendly plant disease control strategies. One promising practice is the utilization of organic amendments, a biologically based protocol, for the control of soil-borne plant pathogens. Plants from the family *Brassicaceae* are usually used as a soil amendment because of their ability to control disease (Lazzeri et al., 2003; Mazzola and Mullinix, 2005; Larkin and Griffin, 2007; Mazzola and Brown, 2010). The mechanism of disease control has been attributed to allelopathy, which is defined as the inhibitory effect of a plant or microorganism (donor) on others (receivers) through the chemicals released from the donor to the environment (Kobayashi, 2004; Hoagland et al., 2008), for example, the glucosinolates within *Brassicaceae*. Glucosinolates are present in every Brassicaceous plant part but are most concentrated in the seed (Borek and Morra, 2005). Following the incorporation of Brassicaceous seed meal (BSM) residues in soil, glucosinolates could be hydrolyzed into a variety of biocidal products, such as isothiocyanates, nitriles, and ionic thiocyanate (SCN^-^), with isothiocyanates being most toxic (Brown and Morra, 1997; Morra and Kirkegaard, 2002). Isothiocyanates have been shown to be highly active in inhibiting a variety of soil-borne plant pathogens, such as *Colletotrichum coccodes, Rhizoctonia solani, Helminthosporium solani* (Taylor et al., 2014), *Phymatotrichopsis omnivora* (Duggar) Hennebert (Hu et al., 2011), and *Aphanomyces euteiches* f. sp. *pisi* (Smolinska et al., 1997).

Brassicaceous plants and BSM residue application have become important tools for disease suppression due to their ability to control unwanted microbial pathogens. However, in the natural environment, rather than living alone, pathogens co-exist/co-live with a diverse microbial community. Non-target microorganisms may also be affected directly or indirectly. In the case of direct impact, the antimicrobial activity of the glucosinolate hydrolysis product is not specific in its mode of action (Scott and Knudsen, 1999), and it is possible that the application of Brassicaceous plant material may adversely affect non-target microorganisms and result in changes in the composition of the non-target microbial community, including those that may be of benefit to the plant or that may play prominent roles in disease suppression. In terms of indirect influence, some non-target microorganisms could compete with the pathogen for space and resource (Lemanceau and Alabouvette, 1993). Once the pathogen is suppressed due to the incorporation of Brassicaceous materials, the non-target microbial microorganisms, including the microbial species that are beneficial for plant growth, may obtain more space and resources, leading to rapid proliferation. Therefore, plant disease control not only indicates pathogen suppression but also implies changes in resource/space distribution and, hence, changes in microbial communities. Understanding the changes in non-target microbial community is important for the ecological assessment of the effect of Brassicaceous plants on soil ecosystems from the viewpoint of microbial ecology. Furthermore, increased understanding is a prerequisite for directing the management of soil-born plant disease through the manipulation of resident microbial communities.

A few studies have been reported on the influence of Brassicaceous plants and BSM residues on the biomass of total bacteria, total fungi, actinomycetes, Gram-negative bacteria, or some specific microbial species, including fluorescent *Pseudomonas* spp., *Streptomyces* spp., and *Pythium* spp. (Scott and Knudsen, 1999; Cohen et al., 2005), in soil through traditional culture-dependent methods. Other authors have documented the influence of isothiocyanates or *Brassicaceae* materials on the overall bacterial or eukaryotic community structure in soil by means of molecular techniques, such as terminal restriction fragment length polymorphism (T-RFLP), denaturing gradient gel electrophoresis (DGGE) (Ibekwe et al., 2001; Rumberger and Marschner, 2003), and phospholipid fatty acid (PLFA) analysis (Ibekwe et al., 2001; Omirou et al., 2011). However, due to the relatively low resolution of these techniques, the soil microbial diversity is far from being understood. Next-generation sequencing could provide not only unprecedented levels of sequence reads to cover and resolve the microbial species (Huse et al., 2008; Hollister et al., 2010), but also relatively finer taxonomic classification information. However, a very limited number of authors have used this method to characterize the soil bacterial and fungal communities after the application of Brassicaceous plant material (Hollister et al., 2013) or isothiocyanates (Hu et al., 2015) in their laboratory microcosm-based (i.e., soil incubation only, without plant and disease assays) studies. Under the condition of plant growth, only one study has used the pyrosequencing technique coupled with a pot-based experiment to depict soil fungal communities under different disease-incidence levels resulting from amendment of the soil with BSM residues (Ma et al., 2015).

*Fusarium* wilt, caused by the soil-borne fungus *Fusarium oxysporum*, is one of the most serious diseases for a series of plants (Yu et al., 2011; Mowlick et al., 2013; Zhao et al., 2014; LaMondia, 2015; Miyatake et al., 2016; Shi et al., 2016). Because of the ability of this wilt pathogen to colonize the roots of a number of weeds and to produce resistant spore structures, it can persist in the soil indefinitely (Yu et al., 2011) and is hence a great threat not only to the quality and quantity of host crops but also to the sustainability of soil ecosystems. Some studies have demonstrated the soil bacterial community responses after the application of Brassicaceous biomass for the purpose of controlling *Fusarium* wilt on spinach (Mowlick et al., 2013) and the soil fungal community responses after BSM residue amendment for the purpose of controlling *Fusarium* wilt on pepper (Ma et al., 2015). However, little is known about the response of soil bacterial communities, especially the rhizosphere bacterial community, to Brassicaceous biomass amendment under the condition that the pepper plant was infected by *Fusarium* wilt pathogen.

In this study, 25-day microcosm incubation of a natural *Fusarium* wilt pathogen-infested soil supplemented with different BSMs was performed. With pepper plants growing in these BSM-treated soils, a 35-day disease assay was further established based on pot experiments. The changes in the bacterial community in the soil after 25 days of incubation and changes in the rhizosphere after an additional 35 days of pepper growth were investigated using 454 pyrosequencing. The objectives of this study were to (1) examine the effects of different BSMs on the bacterial communities and (2) understand the possible relationship between the bacterial community changes resulting from BSM application and *Fusarium* wilt on pepper.

## Materials and Methods

### Soil and Seed Meals

The soil used in this study was collected from the topsoil (0–20 cm) of a commercial chili pepper field in Weslaco, Texas, United States, where *Fusarium wilt* was a consistent and serious problem and where no seed meal amendments were used previously. This soil had a sandy loam texture (sand 69%, silt 14%, and clay 17%) with pH 8.2. The nutrient content of this soil was as follows (Ma et al., 2015): 0.72% organic matter, 10 mg g^-1^ nitrate-N, 125 mg g^-1^ P, and 277 mg g^-1^ K. The soil was passed through a 5-mm mesh before use.

Three types of defatted BSMs, including seed meal of *Camelina sativa* cv. ‘Crantz,’ provided by Dr. Terry Gentry from Texas A&M University, United States; seed meal of *Brassica juncea* cv. ‘Pacific Gold,’ provided by Farm Fuel Inc., Freedom, CA, United States; and seed meal of *Sinapis alba* cv. ‘IdaGold,’ provided by Farm Fuel Inc., Freedom, CA, United States, were used in this study, and these three seed meals are hereinafter called CAME, PG, and IG, respectively. The content and composition of glucosinolate and the content of N, P, and K in these seed meals have been shown previously (Ma et al., 2015). Specifically, PG contained 164 μmol g^-1^ of glucosinolate, with 99% being 2-propenyl (allyl) glucosinolate; IG contained 195 μmol g^-1^ of glucosinolate, with 97% being *p*-hydroxylbenzyl glucosinolate; and CAME contained 23.5 μmol g^-1^ of glucosinolate, with 51.9% being 10-methyl-sulfinyl-decyl-glucosinolate, 30.2% being 11-methyl-sulfinyl-decyl and 17.9% being 9-methyl-sulfinyl-decyl glucosinolate. The N content was 5.9% in CAME, 6.1% in PG, and 6.2% in IG; the P content was 1.01% in CAME, 1.2% in PG, and 1.18% in IG; and the K content was 1.45% in CAME, 1.5% in PG, and 1.37% in IG. These BSMs were sieved through a 1-mm metal mesh before use.

### Soil Incubation and Pot Experiment

Three BSMs, CAME, PG, or a mixture of PG and IG (PG:IG (w/w) = 1:1), were prepared. This preparation resulted in the establishment of three levels of 2-propenyl (allyl) glucosinolate in these seed meals: 0 μmol g^-1^ in CAME, 164 μmol g^-1^ in PG, and 84 μmol g^-1^ in PG+IG. Then, these seed meals were added separately at an application rate of 0.34% (w/w, equivalent to 6000 kg ha^-1^) and mixed thoroughly with the soil. The soil that was amended with no seed meal was used as a control (CK). To bring the N, P, and K content onto a common scale, urea (46-0-0) and compound fertilizer (0-15-15) were added to the CK treatment so that the nutrition level was consistent across all treatments. Soil moisture was then adjusted to approximately 70% of the SWHC (soil water holding capacity) by adding sterilized water. The soil from each treatment was put into three Ziploc^®^ plastic bags, with 7.5 kg soil in each bag. The bags were left open and incubated at room temperature (approximately 24°C) for 25 days. During the incubation period, sterile water was replenished every 2 days to maintain stable soil moisture based on the weight loss. At the end of the incubation period, 10 g soil was collected for DNA extraction after the entire soil in each bag was mixed thoroughly. These incubated soil samples hereinafter are referred to as the pre-planting soil samples.

After collecting pre-planting soil samples, the rest of the soil from each bag was divided into six subsamples of equal weight and then put into six pots. Pepper seedlings (*Capsicum annuum* ‘Ben Villalon,’ 4-leaf stage) were transplanted into the pots at a density of two plants pot^-1^. All the plants grown in these six pots were classified as a replicate group within a treatment. The disease severity of each replicate group was measured at days 7, 15, 25, and 35 after transplanting. The disease severity of pepper was ranked into five levels according to a previous method (Ma et al., 2015): 1, healthy; 2, 1/4 leaves turned yellow and fell off; 3, 1/3 leaves turned yellow and fell off; 4, 1/2 leaves turned yellow and fell off; and 5, dead. The disease index was then calculated according to the formula: Σ(number of diseased plants × corresponding level) ÷ total number of investigated plants × 100. At the end of the pot experiment (35 days after transplanting), rhizosphere soil from each treatment was sampled from three replicate groups for DNA extraction. These samples hereinafter are referred to as the post-planting soil samples. All the pot experiments were conducted in the greenhouse of the Horticulture Department of Texas A&M University.

### Pathogen Characterization

Junctions at 1 cm length between the diseased and healthy tissue on stems of the wilted plants were surface-sterilized and incubated on potato-dextrose agar (PDA) containing 50 μg mL^-1^ rifampicin. When mycelium appeared around the stem, they were further cultured on PDA medium and were identified based on microscopic observation of biological characteristics. *Fusarium* spp. was the only fungus based on the microscopic observation results. To follow Koch’s postulates, this isolate was inoculated in sterilized soil where healthy pepper plants were grown. Compared to the plants in the uninoculated soil, plants in the *Fusarium*-inoculated soil developed the same wilt symptoms. This result further confirmed that the isolated *Fusarium* spp. was the only pathogen.

### DNA Extraction

Total genomic DNA was extracted from 10 g pre-planting soil samples and post-planting soil samples collected after 25 days of incubation and after 35 days of pepper plant growth, respectively, using a PowerSoil DNA Isolation Kit (Mo Bio Laboratories, Inc., Carlsbad, CA, United States) following the manufacturer’s instructions. Extracted DNA was purified using an Illustra MicroSpin S-400HR Column (GE Healthcare Bio-Sciences Corp., Piscataway, NJ, United States). The quality of the purified DNA was examined by electrophoresis in a 0.8% agarose gel. DNA quantity and purity were then determined with a Nanodrop ND-1000 spectrophotometer (NanoDrop Technologies Inc., Wilmington, Delaware, United States). All DNA samples were stored at -20°C until use.

### Real-Time Quantitative PCR

The real-time quantitative PCR technique was applied to quantify the bacterial 16S rRNA gene on a Rotor-Gene 6000 series thermal cycler (Qiagen, Valencia, CA, United States). The forward and reverse primers used were Eub338 (5′- ACT CCT ACG GGA GGC AGC AG-3′) and Eub518 (5′-ATT ACC GCG GCT GCT GG-3′), respectively (Fierer et al., 2005). The standard curve for the bacterial 16S rRNA gene was generated via 10-fold serial dilution of plasmid DNA harboring the target gene. The reaction mixture (15 μL) contained 6.75 μL Real Master Mix with 20 × SYBR solution (5 Prime, Inc., Gaithersburg, MD, United States), 0.5 μL each primer (10 μM), 1.5 μL BSA (10 mg mL^-1^), 1 μL template DNA, and 4.75 μL ddH_2_O. The thermocycling steps were as follows (Fierer et al., 2005): denaturing at 95°C for 15 min; followed by 40 cycles of 1 min at 95°C, 30 s at 53°C, and 1 min at 72°C; and 30 s with a plate read. The negative controls with water as the template instead of soil DNA were always run when the real-time quantitative PCR for soil samples was carried out. Real-time quantitative PCR was performed with three technical replications for each DNA sample.

### 454 Pyrosequencing and Data Analysis

For each soil DNA, the 16S universal bacterial primer set 27F (5′-AGR GTT TGA TCM TGG CTC AG-3′) and 519R (5′-GTN TTA CNG CGG CKG CTG-3′) was used for amplifying the ∼ 500 bp region of 16S rRNA genes. To resolve different samples, an 8-bp barcode was fused to the forward 27F primer. A HotStarTaq Plus Master Mix Kit (Qiagen, Valencia, CA, United States) was used for PCR under the following conditions: 94°C for 5 min, followed by 32 cycles of 94°C for 30 s, 55°C for 30 s, and 72°C for 45 s; and a final elongation step at 72°C for 5 min. The PCR products was visualized and confirmed on a 1.8% agarose gel. All PCR amplicons from different samples were then purified using Agencourt AMPure beads (Agencourt Bioscience Corp., Beverly, MA, United States). The concentration of each purified PCR amplicon was determined using a Nanodrop ND-1000 spectrophotometer (NanoDrop Technologies Inc., Wilmington, Delaware, United States). Equimolar concentrations of the amplicons were then merged into a single tube and subjected to pyrosequencing at Molecular Research Laboratory (Shallowater, TX, United States) using 454 GS FLX titanium technology (454 Life Sciences, Branford, CT, United States). The amplicons were sequenced in the forward direction.

The 454 pyrosequencing data were analyzed using the Quantitative Insights Into Microbial Ecology (QIIME) Pipeline^1^. Specifically, the low-quality sequence reads (reads lengths < 150 bp, ambiguous bases > 0, homopolymers > 6, barcode mismatches, and average quality scores < 25) were discarded, and the 8-bp barcode was examined to distribute the sequences to proper samples. Then, chimeric sequence reads were identified and filtered using the Uchime algorithm based on a chimera-free reference database (Edgar et al., 2011) through the Usearch tool. The operational taxonomic units (OTUs) were generated at a 97% sequence-similarity level (Edgar, 2010). A representative sequence of each OTU was aligned with the PyNAST tool (Caporaso et al., 2010). The taxonomic identification of OTUs was obtained using the RDP Classifier with RDP as the reference database (Wang et al., 2007).

To standardize sampling efforts and to bring the pyrosequences of each sample onto a common scale, all samples were rarefied to an equal sequence depth (866 sequences per sample in this study). The relative abundance of all the phylotypes at each taxonomic level (phylum, class, order, family, and genus) was then summarized. Principal coordinate analysis (PCoA) was performed using the subsampled sequence data to determine the differences in the microbial community structures based on the weighted UniFrac distances, a method that accounts for the phylogenetic relationship between sequences and is thus more powerful than taxon-based measures (Lozupone and Knight, 2005; Hamady et al., 2010). Samples were clustered using UPGMA (unweighted pair group method with arithmetic mean) on weighted UniFrac distances. Three different complementary non-parametric analyses for multivariate data (Zhou et al., 2012), namely, the analysis of similarities (ANOSIM) (Clarke, 1993), non-parametric multivariate analysis of variance using distance matrices (adonis) (Anderson, 2001), and a multi-response permutation procedure (MRPP) (Mielke and Berry, 2001; McCune and Grace, 2002), were used to test for differences in the community structure between treatments. Weighted UniFrac distances were exploited for ANOSIM, Adonis, and MRPP analyses, and a Monte Carlo permutation was used to determine the statistical significance. ANOSIM, adonis, and MRPP were performed using the “vegan” package in the R environment.

For the relative abundance data of each of the taxa, the linear discriminant analysis (LDA) effect size (LEfSe) method^2^ was used to test significant differences between treatments. Furthermore, Pearson correlation analysis was performed to understand the possible relationship between the bacterial taxa and the disease index. The Pearson correlation analysis were processed with the software SPSS.

### Accession Numbers

The original sequence data have been deposited in the European Nucleotide Archive under accession number PRJEB19197.

## Results

### Dynamics of the Plant Disease Severity

**FIGURE 1:**
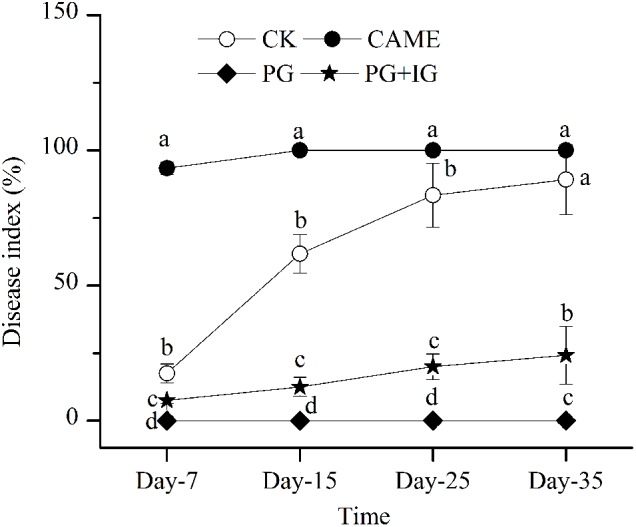
Disease-index dynamics of *Fusarium* wilt on pepper during 35 days of cultivation. The designations CAME, PG, and PG+IG refer to the soils that were amended with different Brassicaceous seed meals (BSMs), namely, *Camelina sativa*, “Pacific Gold”, or both “Pacific Gold” and “IdaGold,” respectively. The designation CK refers to control (no BSM, fertilizer only). The disease severity of pepper was ranked into five levels: 1, healthy; 2, 1/4 leaves turned yellow and fell off; 3, 1/3 leaves turned yellow and fell off; 4, 1/2 leaves turned yellow and fell off; 5, dead. The disease index was then calculated according to the formula Σ(the number of diseased plants × corresponding level) ÷ the total number of investigated plants × 100. The error bar represents the standard deviation of the mean. Different letters indicate significant differences at Duncan’s significance level of 0.05.

Based on the disease index, a parameter used to assess the disease severity, reported on days 7, 15, 25, and 35 after transplanting (**Figure 1**), PG-containing (i.e., PG and PG+IG) treatments significantly (*P* < 0.05) decreased the wilt index compared to the control during 35 days of cultivation (**Figure 1**). PG exhibited the most suppressive effect and completely suppressed the wilt throughout the 35-day period after transplanting. PG+IG treatment also significantly decreased the wilt index by 72.8% (**Figure 1**). However, the addition of CAME did not show an apparent suppressive effect. In addition, a significant negative correlation (*r* = -0.959, *P* = 1.68e-4) was found between 2-propenyl glucosinolate content in BSMs and the disease index on day 35. In other words, the suppressive efficacy was positively correlated with the 2-propenyl glucosinolate contained in the BSMs.

### Bacterial Diversity

**FIGURE 2:**
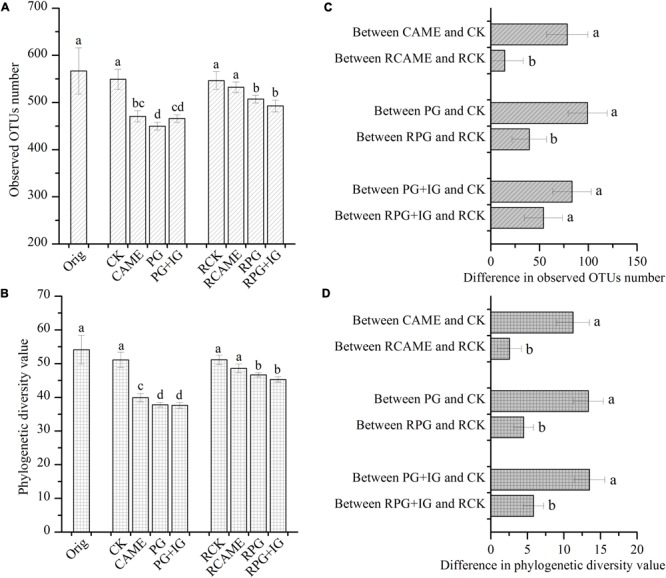
Observed operational taxonomic unit (OTU) number **(A)** and phylogenetic diversity value **(B)** under different treatments and the difference in the observed OTU number **(C)** and phylogenetic diversity value **(D)** between the BSM-amended and the control (CK) treatment. The OTU (at 97% similarity level) number and phylogenetic diversity value were calculated based on subsets of 866 reads per sample. The designation Orig represents the original soil that did not receive any treatment. The designations CAME, PG, and PG+IG represent the soils that were amended with different BSMs, namely, *Camelina sativa*, “Pacific Gold,” or both “Pacific Gold” and “IdaGold”, respectively. The designation CK represents the control (no seed meal, fertilizer only). The designations without the prefix “R” indicate that the samples were collected from the soils that were incubated for 25 days before pepper planting. The designations with the prefix “R” indicate that the soils were collected from the rhizosphere after 35 days of pepper growth. The error bar represents the standard deviation of the mean. Different letters indicate significant differences at Duncan’s significance level of 0.05.

The 16S rRNA gene-based pyrosequencing technique was used to understand the bacterial community changes resulting from BSM amendment and to explore its relationship with plant disease severity. After applying all quality filters, a total of 53,564 high-quality sequences across all samples or an average of 2,060 sequences per sample were obtained (Supplementary Table S1). All BSM treatments significantly decreased the bacterial richness and diversity before pepper planting based on the observation that the observed OTU number and phylogenetic diversity (PD) value were significantly (*P* < 0.05) lower under CAME, PG, and PG+IG treatments in pre-planting soil samples (**Figures 2A,B**). Although the significant decrease still existed after 35 days of pepper growth in PG and PG+IG treatments (**Figures 2A,B**), it was greatly weakened under all BSM treatments, as the difference between all BSM treatments and CK in terms of the observed OTU number and PD value in post-planting soil samples became smaller than that in pre-planting soil samples (**Figures 2C,D**).

Furthermore, after 35 days of pepper growth, in the treatments where the plant disease index was similar, the bacterial richness and diversity were also similar. Specifically, in the CK and CAME soil where the plant disease index was 89.2 and 100, respectively (**Figure 1**), there was no significant difference (*P* > 0.05) in terms of the observed OTU number and the PD value between these two treatments after 35 days of plant growth (**Figures 2A,B**), while in the PG and PG+IG treatments where the plant disease index was obviously decreased but was still similar (PG: 0; PG+IG: 24.2) (**Figure 1**), there was still no significant difference (*P* > 0.05) in terms of the observed OTUs number and the PD value (**Figures 2A,B**). Intriguingly, we also found that 35 days of pepper growth exerted no significant influence on the bacterial richness and diversity when BSM was not amended, while it had significant effect in BSM-amended soil (**Figures 2A,B**).

### Bacterial Community Structure

The bacterial community structure is an overall reflection of the composition and abundance of different taxonomic population in a microbial community. Changes in bacterial community structure as affected by BSMs were thus assessed with the PCoA and UPGMA method based on the weighted UniFrac distances.

**FIGURE 3:**
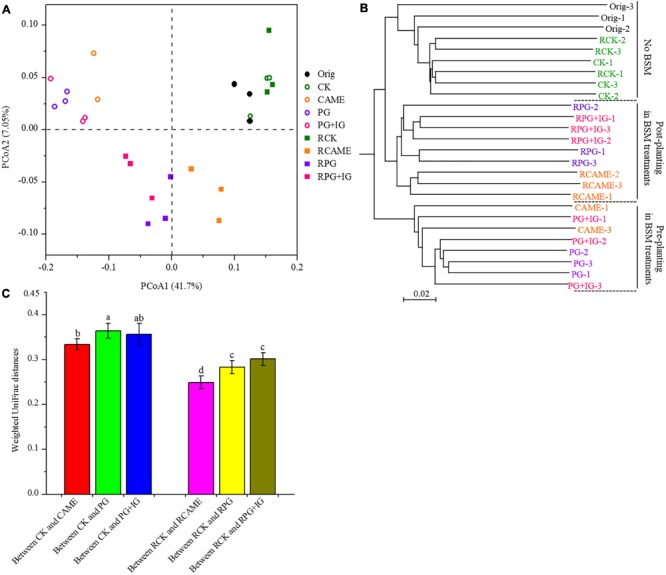
Principal coordinate analysis (PCoA) **(A)** and unweighted pair group method with arithmetic mean (UPGMA) cluster analysis **(B)** of microbial communities and the weighted UniFrac distances of microbial communities between BSM-added and the control (CK) treatment **(C)**. The PCoA plot and UPGMA cluster tree were obtained based on weighted UniFrac distances at a depth of 866 subsampled sequences per sample. In **A**, percentages in parentheses denote the proportions of variation explained by each ordination axis. In **B**, three replicates for each treatment are indicated by the symbols “1,” “2,” and “3.” In **C**, different letters represent significant differences by Duncan’s multiple range test (*P* < 0.05). The error bars indicate the standard deviation of the means. A total of 27 samples were subjected to 454 pyrosequencing, but one sample (CAME-2) only generated 16 good quality sequences, which was exceptional lower than that observed in other samples. The sample CAME-2 was hence not included in the PCoA and UPGMA analyses or in the weighted UniFrac distances calculation. All other designations are the same as those in **Figure 2**.

Twenty-five days of incubation with BSMs significantly changed the bacterial community structure. Specifically, samples from the BSM-amended treatments and unamended control were distributed in different regions of the PCoA space (**Figure 3A**) or grouped in different clusters in the UPGMA tree (**Figure 3B**). Three non-parametric multivariate statistical tests (ANOSIM, adonis, and MRPP) further indicated the significant influence (*P* < 0.05) of the BSMs on the bacterial community structure (**Table 1**). However, the influence of the BSMs on the bacterial community structure was weakened after 35 days of pepper growth based on the observation that the UniFrac distances, a measurement on the dissimilarity in the bacterial community structure, between BSM-amended treatments and CK in the post-planting soil samples were significantly (*P* < 0.05) smaller than in the pre-planting soil samples (**Figure 3C**).

**Table 1:** Significance tests using three statistical approaches to assess the effects of plant growth and BSMs on the overall microbial community structure.

Compared group	adonis^a^		ANOSIM^b^		MRPP^c^	
	
	*F*	*P*^d^	*R*	*P*^d^	*δ*	*P*^d^
Orig vs. CK	1.578	0.107	0.630	0.099	0.326	0.092
Orig vs. RCK	2.790	0.086	0.685	0.106	0.316	0.106
**Plant growth effect**					
CK vs. RCK	1.243	0.107	0.615	0.107	0.291	0.119
CAME vs. RCAME	5.858	**0.029**	0.889	**0.036**	0.292	**0.034**
PG vs. RPG	10.678	**0.032**	0.793	**0.034**	0.251	**0.032**
PG+IG vs. RPG+IG	3.115	**0.037**	0.721	**0.035**	0.268	**0.030**
**BSM effect in pre-planting soil**					
CK vs. CAME	10.822	**0.036**	0.952	**0.034**	0.298	**0.042**
CK vs. PG	34.941	**0.038**	0.943	**0.033**	0.256	**0.032**
CK vs. PG+IG	21.736	**0.036**	0.921	**0.031**	0.287	**0.029**
CAME vs. PG	3.336	0.099	0.502	0.087	0.243	0.101
CAME vs. PG+IG	1.460	0.081	0.523	0.092	0.276	0.126
PG vs. PG+IG	1.921	0.104	0.519	0.084	0.239	0.103
**BSM effect in post-planting soil**					
RCK vs. RCAME	5.072	**0.046**	0.742	**0.042**	0.286	**0.045**
RCK vs. RPG	8.748	**0.040**	0.815	**0.040**	0.288	**0.042**
RCK vs. RPG+IG	16.682	**0.038**	0.895	**0.038**	0.272	**0.034**
RCAME vs. RPG	2.286	**0.048**	0.712	**0.045**	0.290	**0.046**
RCAME vs. RPG+IG	3.751	**0.047**	0.721	**0.044**	0.278	**0.048**
RPG vs. RPG+IG	2.257	0.094	0.663	0.103	0.278	0.113

*All three significant tests are non-parametric multivariate analyses based on dissimilarities among samples. ^*a*^ Permutational multivariate analysis of variance using distance matrices. The significance was obtained with *F*-tests based on sequential sums of squares from permutations of the raw data. ^*b*^ Analysis of similarities. The *R* was obtained based on the difference in the mean ranks between groups and within groups. The significance of observed R was determined by permuting the grouping vector to obtain the empirical distribution of *R* under the null model. ^*c*^ Multi-response permutation procedure. The *δ* is the overall weighted mean of within-group means of the pairwise dissimilarities among sampling units. The significance test refers to as the fraction of permuted *δ* that is less than the observed *δ*. ^*d*^ A Bonferroni correction for the *P* value was applied. Bold number indicates significance at a less than 0.05 level. The designation Orig represents the original soil that did not receive any treatment. The designations CAME, PG, and PG+IG represent the soils that were amended with different Brassicaceous seed meals (BSMs), namely, *Camelina sativa*, “Pacific Gold,” or both “Pacific Gold,” and “IdaGold,” respectively. The designation CK represents the control (no seed meal, fertilizer only). The designations without the prefix “R” indicate that the samples were collected from the soils that were incubated for 25 days before pepper planting. The designations with the prefix “R” indicate that the soils were collected from the rhizosphere after 35 days of pepper growth.*

Furthermore, in the treatments where the plant disease index was similar, the dissimilarity in the bacterial community structure was smaller based on the observation that the UniFrac distances between CAME and CK (**Figure 3C**), where the plant disease index on day 35 was similar (**Figure 1**), was significantly (*P* < 0.05) smaller than that between PG and CK and between PG+IG and CK (**Figure 3C**), where the plant disease index was distant (**Figure 1**) after 35 days of plant growth. Another interesting result was that 35 days of pepper growth had no significant influence on the bacterial community structure when no BSM was added but that it exerted a significant influence on the bacterial community structure in BSM-amended soil (**Figures 3A,B** and **Table 1**).

### Specific Bacterial Population Shifts at the High Taxonomic Level

The changes in some specific bacterial populations resulting from BSM application may be related to differences in plant disease severity. To further understand the specific bacterial population that may be associated with the plant disease severity, the relative abundance of the phylotypes was summarized at the phylum or lower taxonomic level. Then, the correlation between the relative abundance of specific populations at the phylum or lower taxonomic level and observed disease index was analyzed.

**FIGURE 4:**
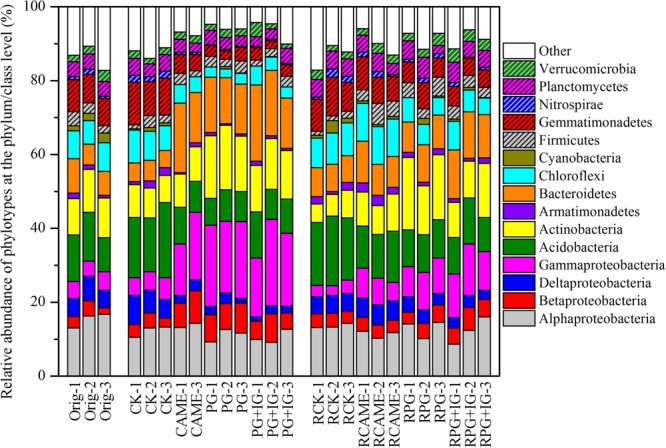
Relative abundance of the phylotypes at the phylum or class (only for Proteobacteria) level. The phylotypes which could be classified at the phylum or class (only for Proteobacteria) level and had relative abundance > 1% in at least on treatment are presented. All other designations are the same as those in **Figures 2, 3**.

**FIGURE 5:**
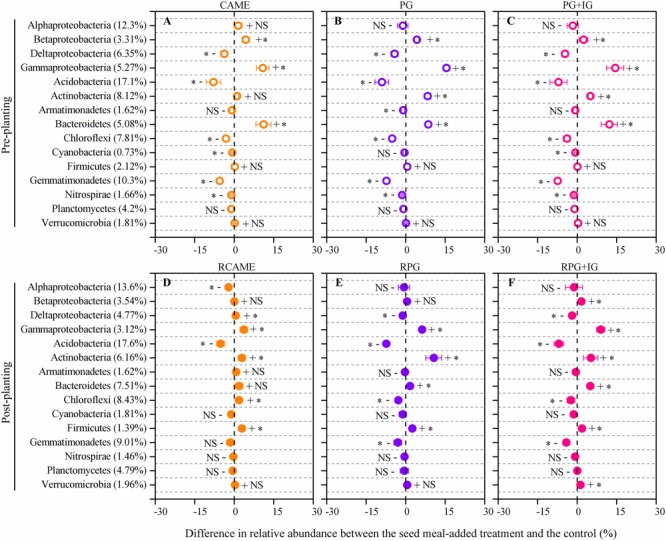
Differences in relative abundance between the BSM-added treatment and the control for those phylotypes presented in **Figure 4**. The net difference in relative abundance was calculated as the relative abundance under BSM-amended treatment – the relative abundance of the phylotypes under control treatment. The LEFSe method was used for testing whether there is a statistically significant difference between the BSM treatments and the control. The LEFSe result is shown in Supplementary Table S2. The error bar denotes the standard deviation of the mean. The percentage in parentheses denotes the relative abundance in the control treatment. The symbols “+” and “-” indicate that the relative abundance was increased or decreased compared with the control. The symbol “^∗^” denotes significant differences at *P* < 0.05; NS denotes no significant difference (*P* > 0.05). All other designations are the same as those in **Figure 2**.

The shift in the relative abundance of the main phyla/classes (those with relative abundance > 1% under at least one treatment were included) are shown in **Figure 4**. Of these main phyla/classes (a total of 15), 12 and 11 phylotypes were significantly changed in relative abundance by at least one BSM treatment in pre-planting soil and post-planting rhizosphere soil samples, respectively (**Figure 5**). Pearson correlation analysis revealed that the relative abundance of Actinobacteria and Gammaproteobacteria in pre-planting soil samples was negatively correlated (*P* < 0.05) with the day-35 disease index, with the correlation being strongest in Actinobacteria (*r* = -0.953, *P* < 0.05), while that of Chloroflexi and Gemmatimonadetes was positively correlated with the day-35 disease index, with the correlation being strongest in Chloroflexi (*r* = 0.760, *P* < 0.05) (**Table 2**). The significant (*P* < 0.05) correlation between these four phylotypes and the day-35 disease index still existed after 35 days of pepper growth, indicating their possible function in manipulating plant disease (**Table 2**). As the bacterial biomass, indicated by the abundance of the 16S rRNA gene copies was not significantly influenced by BSM treatment (Supplementary Figure S1), changes in relative abundance could then reflect the absolute abundance of specific phylotypes.

**Table 2:** Pearson correlation analysis between the relative abundance of phylotypes at the phylum or class (only for Proteobacteria) level and the day-35 plant disease index.

Phylotype	Correlation between the relative abundance of pre-planting phylotype and day-35 disease index		Correlation between the relative abundance of post-planting phylotype and day-35 disease index	
	*r*	*P*	*r*	*P*
Alphaproteobacteria	0.560	0.073	–0.092	0.777
Betaproteobacteria	–0.379	0.250	– 0.507	0.092
Deltaproteobacteria	0.614	0.054	0.828^∗^	0.006
Gammaproteobacteria	–0.736^∗^	0.010	–0.694^∗^	0.018
Acidobacteria	0.580	0.062	0.677^∗^	0.016
Actinobacteria	–0.953^∗^	2.073e-5	–0.797^∗^	0.002
Armatimonadetes	0.443	0.173	0.625	0.030
Bacteroidetes	–0.431	0.186	–0.439	0.153
Chloroflexi	0.760^∗^	0.007	0.871^∗^	0.001
Cyanobacteria	0.270	0.421	0.314	0.321
Firmicutes	–0.227	0.503	–0.224	0.484
Gemmatimonadetes	0.723^∗^	0.012	0.710^∗^	0.010
Nitrospirae	0.699^∗^	0.017	0.336	0.286
Planctomycetes	0.259	0.441	–0.034	0.921
Verrucomicrobia	–0.062	0.857	–0.513	0.088

*The phylotypes shown in **Figure 4** are included here for correlation analyses. The *r* indicates the correlation coefficient. The r that is statistically significant (*P* < 0.05) is indicated by the symbol “^∗^”.*

### Specific Bacterial Population Shifts at a Lower Taxonomic Level

The relative abundance of each phylotype was analyzed at the genus level to understand which bacterial populations were affected by BSM at a finer taxonomic level. Then, the correlation between the relative abundance of the main genera and the disease index was analyzed to explore the possible relationship between bacterial population shifts and plant disease.

**FIGURE 6:**
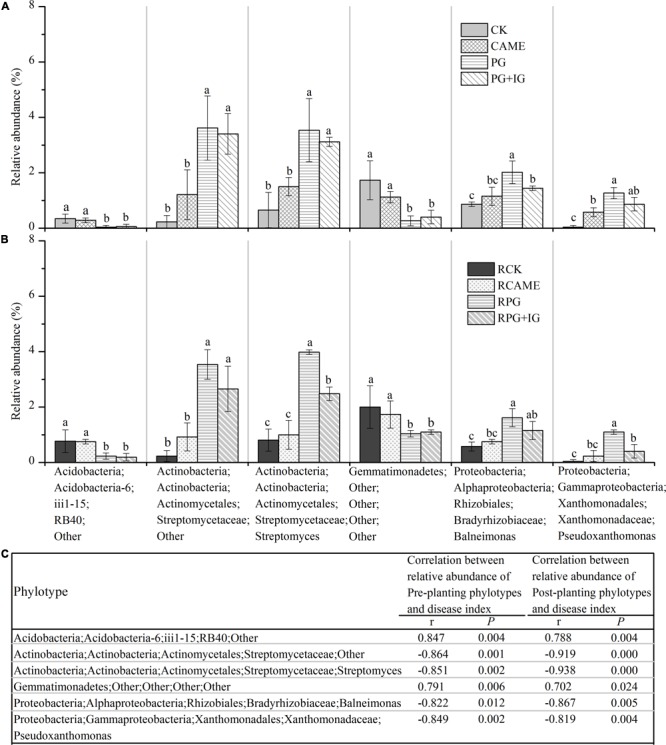
Relative abundance of phylotypes at the genus level **(A,B)** and Pearson correlation analysis between the relative abundance of phylotypes and the day-35 plant disease index **(C)**. Those phylotypes that had relative abundance > 0.5% in at least one treatment and showed significant correlation (*P* < 0.05) with the plant disease index are presented. The r in panel c indicates the Pearson correlation coefficient. The phylotypes are shown in the form of Phylum;Class;Order;Family;Genus. The designation “Other” indicates that the phylotypes are unclassified at the taxonomic level. LEFSe method was used to test significant differences between treatments. The LEFSe results are shown in Supplementary Tables S3, S4. Different letters denote significant differences (*P* < 0.05). The error bar denotes the standard deviation of the mean. All other designations are the same as those in **Figure 2**.

Among the main genera (with relative abundance > 0.5% in at least one treatment), a total of 34 genera was significantly affected by at least one BSM treatment in both pre-planting and post-planting soil samples in terms of relative abundance (**Figures 6A,B** and Supplementary Figure S2). Of these significantly affected genera, the relative abundance of the following 4 genera from pre-planting soil samples, namely, two Streptomycetaceae-affiliated genera within Actinobacteria, i.e., *Streptomyces* and an unclassified genus; the genus *Balneimonas* within Alphaproteobacteria, and the genus *Pseudoxanthomonas* within Gammaproteobacteria, was significantly (*P* < 0.05) increased in the PG and PG+IG treatments (**Figure 6A**) where the plant disease was suppressed, but these genera (except for *Pseudoxanthomonas*) were not affected in CAME treatment (**Figure 6A**) where the day-35 plant disease index was similar to that observed in CK. *Streptomyces* and an unclassified genus within Actinobacteria were the strongest PG- or PG+IG-induced genera in both pre-planting and post-planting soil samples. Pearson correlation analysis further revealed a significantly negative correlation (r ranged from -0.822 to -0.864, *P* < 0.05) between the relative abundance of these 4 phylotypes in pre-planting soil samples and the day-35 plant disease index (**Figure 6C**). This significant correlation still existed after 35 days of pepper growth (r ranged from -0.938 to -0.819, *P* < 0.05). These 4 genera were hereinafter called significantly and negatively related phylotypes. In contrast, the relative abundance of the other 2 genera from pre-planting soil samples, namely, an unclassified genus within Acidobacteria and an unclassified genus within Gemmatimonadetes, was significantly decreased in PG-containing treatments (i.e., PG and PG+IG), but their relative abundance was not affected in the CAME treatment (**Figure 6B**). Pearson correlation analysis further indicated a significantly positive correlation (*r* = 0.847 for the Acidobacteria-affiliated genus, *r* = 0.791 for the Gemmatimonadetes-affiliated genus; *P* < 0.05) between the relative abundance of these 2 pre-planting phylotypes and the day-35 plant disease index (**Figure 6C**). The significant correlations still existed after 35 days of pepper growth (*r* = 0.788 for the Acidobacteria-affiliated genus, *r* = 0.702 for the Gemmatimonadetes-affiliated genus; *P* < 0.05). These 2 genera were hereinafter called significantly and positively related phylotypes. As no significant changes were found in terms of total bacterial biomass, as assessed by quantifying the 16S rRNA gene using real-time quantitative PCR, under BSM treatments (Supplementary Figure S1), relative abundance data could then reflect the changes in the absolute abundance of specific taxa.

*Streptomyces* and an unclassified genus within Actinobacteria were the strongest PG- or PG+IG-induced genera in both pre-planting and post-planting soil samples. The unclassified genus within Actinobacteria exhibited approximately 15- and 14-fold increases in relative abundance within 25 days of PG and PG+IG amendment, respectively, with its relative abundance increasing from 0.23% under CK to 3.62% and 3.41% under PG and PG+IG treatments, respectively (**Figure 6A**). This elevated relative abundance was maintained after 35 days of pepper growth, with its relative abundance increasing from 0.23% under CK to 3.54% under PG and 2.65% under PG+IG (**Figure 6B**). Similarly, the Actinobacteria-affiliated *Streptomyces* showed a significant increase (approximately a 2–4 times increase) in its relative abundance in PG and PG+IG in pre-planting and post-planting soil samples, with its relative abundance increasing from 0.65-0.80% under CK to 3.54–3.98% and 2.48–3.11% under PG and PG+IG treatments, respectively, in pre-planting and post-planting soil samples (**Figures 6A,B**). Correlation analysis further revealed that the relative abundance of these two phylotypes in both pre-planting and post-planting rhizosphere soil samples showed the strongest relationship with the day-35 plant disease index (**Figure 6C**).

## Discussion

Plant-disease suppression by Brassicaceous biomass has long been attributed to glucosinolate-induced antimicrobial effects. Previous studies have shown that the inhibitory efficacy of isothiocyanate for plant diseases/plant pathogens varies depending on its structure and concentration (Aires et al., 2009; Taylor et al., 2014). It is therefore suspected that the content and composition of glucosinolate (the precursor of isothiocyanates) added within BSMs could influence the plant disease severity. Indeed, we found that it was the content of 2-propenyl (allyl) glucosinolate (within PG), or the capacity of the glucosinolate to produce 2-propenyl (allyl) glucosinolate, rather than 10-methyl-sulfinyl-decyl-glucosinolate (within CAME), that was negatively associated with disease severity. For instance, in the PG and PG+IG treatments with 2-propenyl (allyl) glucosinolate, the wilt disease was significantly suppressed, while in the CAME treatment with no 2-propenyl (allyl) glucosinolate, the disease index developed immediately to a high level. The phenomenon may be attributed to the proliferation or suppression of the *Fusarium* wilt pathogen resulting from treatment with the different BSMs. Our previous work (Ma et al., 2015), which focused on fungal communities after BSM incorporation based on a different run of the experiment (although the design follows that in this study), found that the growth of *Fusarium* was suppressed in PG-containing treatments, while it was promoted under a CAME treatment, and this finding supported our speculation.

In addition to the antimicrobial effects of 2-propenyl (allyl) glucosinolate within applied BSMs leading to the difference in plant disease severity, the changes in bacterial communities resulting from BSM incorporation may be one of the microbial mechanisms contributing to variation in plant disease severity. At a course taxonomic level, Chloroflexi, the phylum that showed the strongest positive correlation with the disease index in our study has been regarded as disease-inducible bacteria (Niu et al., 2016) and has been found to be positively correlated with the morbidity of tobacco bacterial wilt (Niu et al., 2016) and cucumber *Fusarium* wilt (Li et al., 2016). Actinobacteria, which has been identified as the most dynamic phylum in a soil suppressive to the fungal root pathogen *Rhizoctonia solani* (Cordovez et al., 2015), was also found to have the strongest negative correlation with the disease index in our study. Similar to our finding, higher relative abundance of Actinobacteria was also found in a rhizosphere soil where the incidence of watermelon *Fusarium* wilt was decreased (Xu et al., 2015). We suspect that the proliferation of beneficial bacterial populations such as Actinobacteria and the suppression of unfavorable phylotypes for plant disease, such as Chloroflexi was one of the important microbial mechanisms contributing to the wilt suppression in PG-containing treatments.

Furthermore, a close relationship between bacterial population shifts and plant disease severity was found at a finer taxonomic level. The Actinobacteria-affiliated unclassified genus and *Streptomyces* were the strongest PG- and PG+IG-induced genera, with their relative abundance increased by 14–15-fold for the unclassified genus and by 3.8–4.4-fold for *Streptomyces* after 25 days of incubation. This increase in relative abundance was maintained after an additional 35 days of pepper growth. These two phylotypes also showed the strongest negative correlation with the plant disease index. Actinobacteria, and specifically Streptomycetaceae, have been found to contain a number of strains contributing to disease-suppressive soils and antifungal activities (Viaene et al., 2016). *Streptomyces*, the largest genus in Actinobacteria (Viaene et al., 2016), has been shown to suppress a range of plant pathogens (Castillo et al., 2002; Cohen et al., 2005) through producing antibiotics (Lucas et al., 2013) and other bioactive metabolites, including volatile organic compounds (Cordovez et al., 2015) and cell wall-degrading enzymes, such as chitinases (Hoster et al., 2005). The ability of *Streptomyces* strains to suppress plant diseases, such as *Rhizoctonia solani* damping-off on tomato and *Fusarium* wilt on chickpea caused by *Fusarium oxysporum*, has been confirmed through greenhouse and field studies (Gopalakrishnan et al., 2011; Goudjal et al., 2014). Therefore, the disease suppression by PG-containing treatments in our study may be explained by the significant increase in Actinobacteria-affiliated genera by PG and PG+IG treatments, which also contributed to the inhibition of the growth of the *Fusarium* wilt pathogen and hence the decrease in the severity of *Fusarium* wilt on pepper. Indeed, the suppression of *Fusarium* was found in PG-containing treatments where the wilt disease was suppressed greatly in our previous study based on another experimental run (Ma et al., 2015). For the positively correlated genera, i.e., the RB40-affiliated genus within Acidobacteria and an unclassified genus within Gemmatimonadetes, because both of them cannot be classified at the genus level, it is difficult to relate their biological properties to plant disease. It is likely that these two genera formed cooperative relationships with the plant pathogen, leading to severe plant disease, although the mechanisms behind this process remain to be clarified. To some extent, this speculation was supported by an *in silico* approach-based study (da Silva et al., 2014), which found that the non-pathogenic bacterial species (e.g., *Erwinia toletana*) and the pathogen (*Pseudomonas savastanoi* pv. *savastanoi*) formed a cooperative interaction via quorum-sensing signal-sharing, and the outcome of this interaction was a more aggressive knot disease on olive when co-inoculations were made compared with single inoculations.

BSM addition decreased the bacterial richness and diversity before pepper planting and altered the bacterial community structure in both pre-planting soil and post-planting rhizosphere soil. The decrease in the bacterial richness and diversity was similar to the observations in a soil microcosm-based study (Hollister et al., 2013), which found that the addition of glucosinolate-containing BSM resulted in 38.8% and 22.0% decreases in the OTU number and Shannon index of the bacterial community, respectively, after 28 days of incubation. Another field trial-based study (Wei et al., 2016) also demonstrated a decrease in the OTU number and Shannon index in soil treated with *Brassica* seed meal. Similar to our finding, significant changes in the bacterial community structure resulting from BSM incorporation was also found in previous studies (Hollister et al., 2013). The changes in the bacterial community structure may be due to the fact that glucosinolates and their breakdown products could act as a factor of selection on soil microbial communities. To some extent, this speculation was supported by a previous study (Bressan et al., 2009) showing that glucosinolate and its hydrolysis product could have important repercussions for soil microbial communities in the rhizosphere and that even minor modification in the profile of glucosinolate produced by the root of *Arabidopsis thaliana* significantly influenced the microbes in the rhizosphere and plant roots.

Intriguingly, we showed that the original soil was more easily affected by the BSMs than by 35 days of pepper growth based on the phenomenon that 35 days of pepper growth exerted no significant influence on the bacterial richness, diversity, and structure when BSM was not amended, while it showed a significant effect on BSM-amended soil. Once the bacterial community in the original soil was disturbed by the BSMs, the 35 days of pepper growth tended to help the bacterial communities to recover from the disturbed state to be more similar to the original state. In other words, the effect of BSMs on the bacterial community was weakened by 35 days of pepper growth. However, in our previous study (which reported a different run of the experiment, although the design follows that in this study) (Ma et al., 2015), unlike bacterial communities, BSM application altered the fungal community structure, but the fungal community did not show any indication of resilience (i.e., returned to their nonamended control state) after pepper growth. In other words, the effect of 35 days of pepper growth on the fungal community was minor. This observation may be because of fungi being more sensitive to BSMs than bacteria. A previous study has shown that the greatest effects of BSM addition occurred within the fungal portion of soil communities, although both bacterial and fungal communities were affected by BSM amendment (Hollister et al., 2013), and this finding supported our speculation. Similar to our finding, Hollister et al. (2013) revealed that *Brassica juncea* seed meal treatment altered soil fungal communities dramatically and that its influence could be sustained for a longer time than those imparted upon the bacterial community.

Furthermore, the finding that once disturbed by BSMs, the bacterial community tended to shift back toward the prior state after 35 days of pepper growth may be explained by the fact that the soil sample used in this study was collected from a commercial chili pepper field where *Fusarium wilt* was a consistent and serious problem due to consistent planting, so that the bacteria formed a relatively stable structure to make themselves adaptable to continuous cropping; however, BSMs had never been used in that field. These observations may explain why the bacterial community structure in the original soil was more easily affected by the sudden BSM incorporation than by the usual and “already accustomed to” practice, namely, planting. Similarly, this may also explain why in the case when the bacterial community was changed in terms of the bacterial richness, diversity, and structure, it tended to recover to its original state after pepper planting. The paramount role of further pepper planting in recovering the bacterial communities in this study explained one of the reasons why continuous cropping usually makes the soil unfavorable for plant growth.

In summary, the suppression of *Fusarium* wilt on pepper was achieved by application of PG-containing BSMs. In addition to wilt suppression, the PG-containing BSM addition also altered the bacterial community structure and decreased the bacterial richness and diversity. Pepper planting could weaken the BSM effect on the bacteria communities. Besides through chemical/biocidal action of 2-propenyl (allyl) glucosinolate within applied BSMs that suppressed *Fusarium* wilt on pepper, the changes in bacterial populations at the phylum/class and genus levels resulting from PG-containing BSM treatments, such as the significant increase in Actinobacteria-affiliated genera, very common disease-suppressive phylotypes, and the significant decrease in Chloroflexi, a reported disease-inducible population (Niu et al., 2016), are suspected to be one of the microbial mechanisms that are involved in PG-containing BSM-induced disease suppression. Further studies should focus on evaluating the contribution of specific taxa (such as Actinobacteria-affiliated genera) to disease suppression *in situ* and testifying the viability and effectiveness of the method of manipulation of the resident bacterial taxa (e.g., Actinobacteria-affiliated genera) for plant disease control.

## Author Contributions

GR contributed to conception, data analysis, and interpretation and drafted and critically revised the manuscript. YM contributed to conception, design, data acquisition, and interpretation and critically revised the manuscript. DG, TG, PH, EP, and MG contributed to conception and critically revised the manuscript.

## Conflict of Interest Statement

The authors declare that the research was conducted in the absence of any commercial or financial relationships that could be construed as a potential conflict of interest.

## Footnotes

1 http://qiime.org/

2 http://huttenhower.sph.harvard.edu/galaxy/
