# Supplementary material for: Soil Bacterial Community Was Changed after Brassicaceous Seed Meal Application for Suppression of Fusarium Wilt on Pepper
Source: Front Microbiol. 2018 Feb 13;9:185. doi: 10.3389/fmicb.2018.00185 (PMC5816756; doi:10.3389/fmicb.2018.00185)
Supplement: Supplementary file 1 [file Image_1.PDF]

Table S1. Number of high-quality reads.

Sample	High quality reads number
Orig	2,103 ±1,319
CK	2,073 ±236
CAME	1,104 ±980
PG	1,830 ±956
PG+IG	2,526 ±237
RCK	1,973 ±241
RCAME	2,009 ±345
RPG	1,815 ±641
RPG+IG	2,427 ±167
Total	53,564
Average	2,060

The designations CAME, PG, and PG+IG denote the soils that were amended with different Brassicaceous seed meals (BSMs), namely, *Camelina sativa*, "Pacific Gold", or both “Pacific Gold” and "IdaGold", respectively. The designation CK denotes the control (no BSM, fertilizer only). The designations without the prefix "R" indicate that the samples were collected from the soils that were incubated for 25 days without pepper planting. The designations with the prefix "R" indicate that the soils were collected from the rhizosphere after 35 days of pepper growth. The designation Orig denotes the original soil that did not receive any treatment. The data presented are the mean values of three replicates ± the standard deviation.

Table S2. Linear discriminant analysis (LDA) effect size (LEfSe) results on relative abundance of phylotypes at the phylum/class level.

Phylotype	CK vs. CAME		CK vs. PG		CK vs. PG+IG		RCK vs. RCAME		RCK vs. RPG		RCK vs. RPG+IG	
	LDA score	*P*[a]	LDA score	*P*[a]	LDA score	*P*[a]	LDA score	*P*[a]	LDA score	*P*[a]	LDA score	*P*[a]
Alphaproteobacteria	4.000	0.264	3.882	0.310	4.008	0.310	4.127	0.049	3.911	0.827	3.974	0.513
Betaproteobacteria	4.423	0.043	4.459	0.042	4.194	0.047	3.769	0.827	3.909	0.354	3.942	0.048
Deltaproteobacteria	4.297	0.049	4.335	0.040	4.368	0.036	3.697	0.047	4.224	0.043	4.240	0.026
Gammaproteobacteria	4.777	0.039	4.959	0.023	4.883	0.031	4.299	0.032	4.647	0.027	4.677	0.028
Acidobacteria	4.550	0.043	4.671	0.026	4.581	0.036	4.428	0.039	4.612	0.019	4.555	0.028
Actinobacteria	4.021	0.109	4.682	0.023	4.515	0.025	4.150	0.040	4.740	0.031	4.422	0.049
Armatimonadetes	3.990	0.264	4.166	0.049	4.054	0.127	3.806	0.330	3.948	0.543	3.834	0.310
Bacteroidetes	4.781	0.039	4.631	0.020	4.760	0.030	4.129	0.199	4.273	0.049	4.526	0.024
Chloroflexi	4.379	0.040	4.502	0.019	4.356	0.029	4.134	0.031	4.296	0.036	4.091	0.049
Cyanobacteria	3.711	0.049	4.064	0.152	3.973	0.046	3.870	0.199	3.961	0.354	3.985	0.174
Firmicutes	4.364	0.805	3.622	0.543	3.550	0.827	4.211	0.043	4.239	0.046	4.143	0.034
Gemmatimonadetes	4.491	0.035	4.596	0.029	4.595	0.032	3.928	0.330	4.249	0.049	4.375	0.038
Nitrospirae	3.972	0.043	4.207	0.027	4.166	0.030	3.550	0.330	4.015	0.543	3.907	0.310
Planctomycetes	4.059	0.264	3.823	0.152	3.855	0.152	3.778	0.543	3.912	0.543	3.829	0.553
Verrucomicrobia	3.767	0.564	3.966	0.827	3.825	0.827	3.697	0.543	4.012	0.354	3.992	0.049

Those phylotypes shown in Figure 5 are included for LEFSe analysis. [a] The *P* values was adjusted with the False Discovery Rate approach. All other designations are the same as those in Table S1.

Table S3. Linear discriminant analysis (LDA) effect size (LEfSe) results on relative abundance of phylotypes from pre-planting soil samples at the genus level.

Phylotype	CK vs. CAME		CK vs. PG		CK vs. PG+IG		CAME vs. PG		CAME vs. PG+IG		PG vs. PG+IG	
	LDA score	*P* [a]	LDA score	*P* [a]	LDA score	*P* [a]	LDA score	*P* [a]	LDA score	*P*	LDA score	*P* [a]
Acidobacteria;Acidobacteria-6;iii1-15;RB40;Other	2.926	0.305	3.260	0.046	3.224	0.049	3.401	0.046	3.042	0.046	2.297	0.684
Actinobacteria;Actinobacteria;Actinomycetales;Streptomycetaceae;Other	3.524	0.128	4.023	0.041	4.041	0.044	3.908	0.048	3.791	0.047	3.095	0.827
Actinobacteria;Actinobacteria;Actinomycetales;Streptomycetaceae;Streptomyces	3.378	0.574	3.949	0.042	3.892	0.039	3.723	0.043	3.789	0.042	3.098	0.684
Gemmatimonadetes;Other;Other;Other;Other	3.442	0.128	3.818	0.043	3.783	0.045	3.534	0.044	3.473	0.044	2.826	0.612
Proteobacteria;Alphaproteobacteria;Rhizobiales;Bradyrhizobiaceae;Balneimonas	3.914	0.128	3.502	0.046	3.230	0.047	3.412	0.048	3.987	0.196	3.145	0.049
Proteobacteria;Gammaproteobacteria;Xanthomonadales;Xanthomonadaceae; Pseudoxanthomonas	3.313	0.046	3.657	0.043	3.520	0.044	3.321	0.043	3.054	0.248	3.049	0.330

Those genera shown in Figure 6 are included for testing. [a] The *P* values was adjusted with the False Discovery Rate approach. All other designations are the same as those in Table S1.

Table S4. Linear discriminant analysis (LDA) effect size (LEfSe) results on relative abundance of phylotypes from post-planting rhizosphere soil samples at the genus level.

Phylotype	RCK vs. RCAME		RCK vs. RPG		RCK vs. RPG+IG		RCAME vs. RPG		RCAME vs. RPG+IG		RPG vs. RPG+IG	
	LDA score	*P* [a]	LDA score	*P* [a]	LDA score	*P* [a]	LDA score	*P* [a]	LDA score	*P*	LDA score	*P* [a]
Acidobacteria;Acidobacteria-6;iii1-15;RB40;Other	2.645	0.569	3.418	0.048	3.438	0.048	3.418	0.046	3.415	0.046	2.507	0.605
Actinobacteria;Actinobacteria;Actinomycetales;Streptomycetaceae;Other	3.370	0.390	4.047	0.038	3.918	0.041	3.956	0.043	3.786	0.049	3.391	0.374
Actinobacteria;Actinobacteria;Actinomycetales;Streptomycetaceae;Streptomyces	2.965	0.827	4.014	0.038	3.723	0.042	4.017	0.042	3.661	0.043	3.778	0.046
Gemmatimonadetes;Other;Other;Other;Other	3.222	0.569	3.766	0.046	3.755	0.048	3.488	0.049	3.476	0.049	2.419	0.605
Proteobacteria;Alphaproteobacteria;Rhizobiales;Bradyrhizobiaceae;Balneimonas	2.916	0.227	3.497	0.044	3.196	0.047	3.418	0.048	3.123	0.108	3.365	0.195
Proteobacteria;Gammaproteobacteria;Xanthomonadales;Xanthomonadaceae; Pseudoxanthomonas	2.860	0.390	3.590	0.042	3.138	0.046	3.490	0.044	2.844	0.827	3.398	0.048

Those genera shown in Figure 6 are included for testing. [a] The *P* values was adjusted with the False Discovery Rate approach. All other designations are the same as those in Table S1.

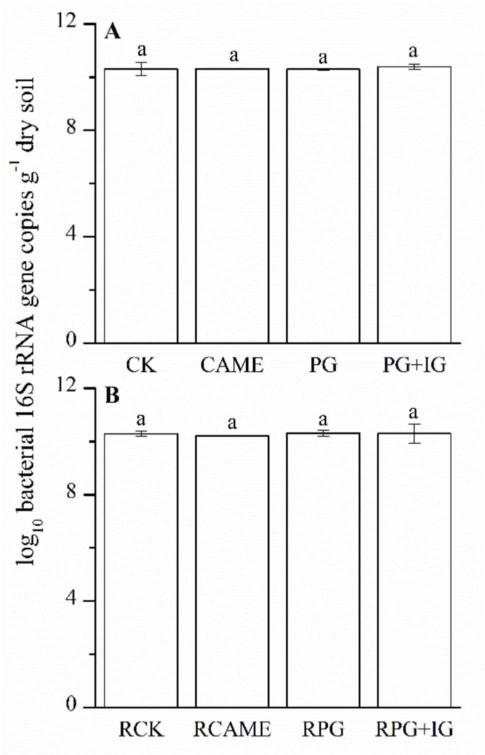

Figure S1. Quantification of the bacterial 16S rRNA gene in pre-planting soil (A) and post-planting rhizosphere soil (B) using real-time quantitative PCR. The results are expressed as $\log_{10}$ bacterial 16S rRNA gene copies g^{-1} dry soil. The same letters are not significantly different at Duncan's significance level of 0.05. The error bar represents the standard deviation of the mean. All other designations are the same as those in Table S1.

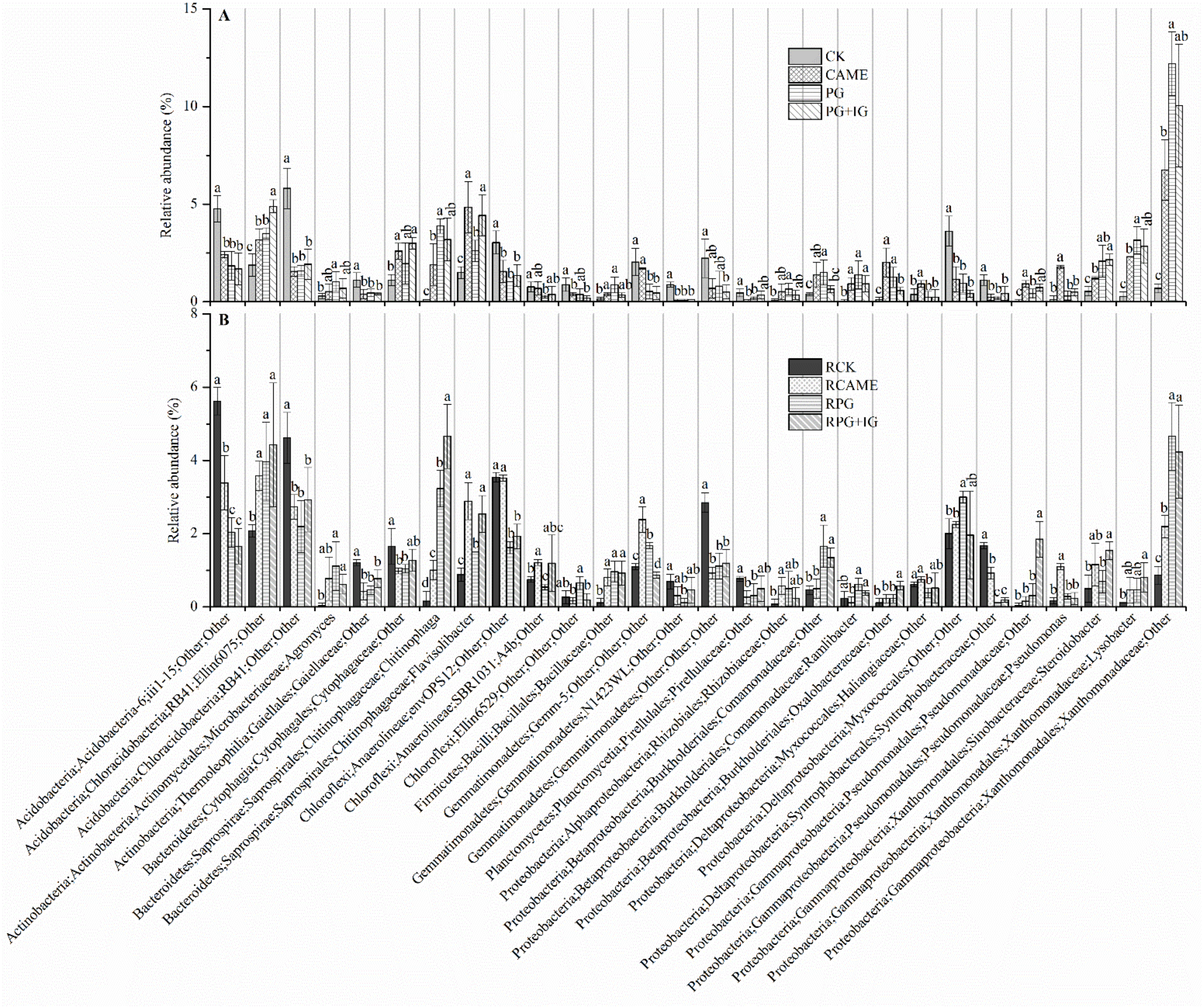

Figure S2. Relative abundance of phylotypes from pre-planting soil (A) and post-planting rhizosphere soil (B) at the genus level. Those phylotypes that had a relative abundance >0.5% in at least one treatment are presented. The phylotypes are shown in the form of Phylum;Class;Order;Family;Genus. The designation "Other" indicates that the phylotype are unclassified at the taxonomic level. LEfSe method was used to test significant differences between treatments. Different letters represent significant differences (P<0.05). The error bar represents the standard deviation of the mean. All other designations are the same as those in Table S1.
